# A study on the differences in recovery effects of different types of nutritional supplements on competitive performance of esports athletes under mental fatigue

**DOI:** 10.1080/15502783.2026.2728354

**Published:** 2026-09-09

**Authors:** Yiyuan Chong, Mengli Wei, Jun Tu, Ziyang Li, Minghui Li, Yaping Zhong

**Affiliations:** a School of Sports Training, Wuhan Sports University, Wuhan, China

**Keywords:** Esports, heart rate variability, mental fatigue, nutritional supplements

## Abstract

**Background:**

To compare the effects of different nutritional supplements on the recovery of core competitive performance abilities in esports athletes following mental fatigue and to observe changes in the autonomic nervous system during recovery after nutritional supplementation by monitoring heart rate variability (HRV).

**Methods:**

A randomized crossover within-subject controlled experimental design was adopted, including nutritional supplement type (caffeine, nitrate, Ginkgo biloba extract, catechins, placebo) × mental fatigue state (initial, fatigued, post-supplementation). Twenty high-level first-person shooter (FPS) esports athletes were recruited. Mental fatigue was induced using a Stroop task. After ingesting the different supplements and resting for 60 minutes, the participants completed assessments of shooting accuracy, shooting stability, spatial localization, and multitasking ability using the KovaaK's simulation trainer. HRV indices were also recorded to evaluate changes in autonomic regulation.

**Results:**

For shooting accuracy, compared with the placebo condition, all four supplements significantly improved shooting accuracy scores following mental fatigue (all *p *< 0.05); however, no significant differences were observed among the effects of the different supplements. For shooting stability, caffeine, nitrate, and catechins produced significant recovery effects on shooting stability (all *p* *< *0.05); however, no significant differences were observed among the effects of these three supplements. For spatial localization and multitasking ability, the improvements in these two abilities in the post-supplementation state may have resulted from natural recovery, and none of the four nutritional supplements demonstrated a significant recovery effect. The HRV results showed that indices including RMSSD and SDNN changed under some supplement conditions.

**Conclusions:**

Mental fatigue significantly reduced the competitive performance of esports athletes. Four types of nutritional supplements all promoted the recovery of shooting accuracy, while caffeine, nitrate, and catechins promoted the recovery of shooting stability. However, no additional recovery advantages of the nutritional supplements over placebo were identified for spatial localization or multitasking ability. Changes in HRV may reflect changes in autonomic regulation during recovery, but further research is still warranted.

## 1 Introduction

In recent years, major international esports events, such as the esports competition at the Hangzhou Asian Games and the Esports World Cup in Riyadh, Saudi Arabia, have been held worldwide. This development marks the formal emergence of esports onto the central stage of global competitive sport and places greater demands on the competitive performance of esports athletes [1]. Esports performance is highly dependent on sustained, high-intensity cognitive activity. Athletes must maintain accurate aiming, recoil control during sustained fire, and multitarget decision-making throughout training sessions or competitions lasting 5–10 hours. Such prolonged periods of heightened neural activation can readily induce mental fatigue [2–4].

Esports performance encompasses multiple dimensions, including shooting accuracy, shooting stability, spatial localization, and multitasking ability. Mental fatigue can impair these core cognitive abilities and ultimately exert a direct negative effect on competitive outcomes [5–8]. Therefore, identifying effective strategies for alleviating mental fatigue is essential for esports athletes to maintain optimal competitive performance.

Nutritional supplementation is an established strategy for alleviating fatigue in conventional sports, and its potential application in esports has attracted increasing attention. The cognitive-enhancing effects of caffeine have been widely demonstrated. By antagonizing central adenosine A1 receptors and enhancing cholinergic neurotransmission, caffeine may improve attention, reaction ability, and various aspects of athletic performance [9–14]. However, the effects of caffeine on complex cognitive–motor tasks may depend on dosage, individual caffeine sensitivity, and task type, and consistent improvements have not been observed across all cognitive tasks [15]. Nitrate can be converted into nitric oxide, thereby improving cardiorespiratory endurance [16]. By increasing regional cerebral blood flow within the frontal white matter, particularly between the anterior cingulate cortex and the dorsolateral prefrontal cortex, nitrate may enhance perfusion in brain regions associated with executive function at rest. Nitrate supplementation may also increase cerebral blood flow during exercise and reduce mental fatigue [17,18]. However, findings from existing clinical studies regarding the cognitive effects of nitrate remain inconsistent. These effects may be influenced by factors such as intervention duration, participants' health status, and the methods used for cognitive assessment [19]. The effects of Ginkgo biloba extract on the cognitive, physiological, and psychological sequelae associated with neurological and vascular disorders have been widely investigated. The flavonoid components of Ginkgo biloba extract may inhibit cortisol release and influence reaction time, attention, psychomotor function, fatigue, mood, performance outcomes, and information-processing speed [20–22]. However, existing findings are not entirely consistent, and the effects may be influenced by differences in extract standardization, dosage, study population, and cognitive assessment methods [23]. Catechins have demonstrated substantial antioxidant, anticancer, anti-inflammatory, and antimicrobial properties in numerous human, animal, and in vitro studies [24]. Previous studies have shown that catechins can improve working memory [25] and enhance attention during complex tasks [26–29]. However, some studies have not found evidence that catechins can modulate cognitive performance [30].

Although previous studies have indicated that different nutritional supplements may positively affect cognitive function and athletic performance, systematic comparative research on recovery from mental fatigue in esports athletes remains limited. In particular, within esports environments characterized by high cognitive load and substantial demands for fine motor control, it remains unclear whether different nutritional supplements can provide additional benefits beyond the natural recovery process. Therefore, clarifying the effects of different nutritional supplements on the recovery of esports-specific abilities may not only enrich the theoretical foundations of mental-fatigue recovery within sports nutrition but also provide a scientific basis for developing individualized nutritional recovery strategies for esports athletes.

Accordingly, this study employed a randomized crossover within-subject controlled design incorporating nutritional supplement type (caffeine, nitrate, Ginkgo biloba extract, catechins, and placebo) × mental fatigue state (initial, fatigued, and post-supplementation). The study aimed to systematically compare the effects of different nutritional supplements on the recovery of esports-specific performance following mental fatigue and to determine whether the supplements exhibited different levels of recovery-promoting potential. In addition, heart rate variability (HRV) was monitored dynamically to assess changes in autonomic nervous system regulation during supplementation and to explore the potential physiological mechanisms through which nutritional supplements may facilitate recovery from mental fatigue. This study may deepen the understanding of recovery from cognitive fatigue in esports athletes, extend the theoretical basis of sports nutrition in competitive settings characterized by high cognitive demands, and provide a theoretical foundation for developing scientific and individualized nutritional recovery strategies and optimizing the management of training and competition status in esports athletes.

Two hypotheses were proposed. First, compared with the mentally fatigued state, esports performance would recover to some extent following a 60-minute recovery period, and nutritional supplementation might further modulate this recovery process, with different supplements potentially producing different recovery effects. Second, nutritional supplementation might affect autonomic regulation during recovery, as reflected by changes in HRV indices, including the root mean square of successive differences (RMSSD), standard deviation of normal-to-normal intervals (SDNN), high-frequency power (HF), and the low-frequency to high-frequency power ratio (LF/HF).

## 2 Methods

### 2.1 Participants

The required sample size was estimated using G*Power version 3.1.9.7. Based on a previous study [31], the effect size was set at 0.6, with a significance level of *α* = 0.05 and statistical power of 0.80. The calculation indicated that a minimum sample of 15 participants was required. After accounting for a potential dropout rate of 20%, 20 high-level first-person shooter (FPS) esports athletes were ultimately recruited.

All participants were required to meet the following inclusion criteria: (1) healthy adults without psychiatric disorders, insomnia, or other abnormal mental health conditions [32]; and (2) at least two years of experience playing FPS games, at least 30 hours of online gameplay per week [33], and attainment of a high competitive rank, defined as Ascendant or above in Valorant or Grade A or above on the 5E Arena platform for Counter-Strike: Global Offensive/Counter-Strike 2. Participants' competitive rankings were verified using their game account profiles or screenshots displaying their rankings. Based on the definition proposed by Formosa et al. [34], which characterizes esports as organized, competitive digital gaming activities, participants who met the above criteria for FPS experience, weekly training duration, and competitive ranking were classified as high-level FPS esports athletes.

The exclusion criteria were as follows: (1) insufficient gaming experience; (2) allergy to caffeine supplements [35]; and (3) the presence of circulatory system disorders, such as cardiac arrhythmias, or current medication use [36]. Basic information of the subjects is shown in Table 1. The study was approved by the Medical Ethics Committee of Wuhan Sports University (approval no. 2025140).

**Table 1.** Subject's basic information.

N(M/F)	Age	Height (cm)	Weight (kg)	Weekly training duration (h)	Game duration (year)
20(18/2)	21 ± 2.8	178.6 ± 7.23	76.22 ± 6.31	37.2 ± 8.6	≥2

### 2.2 Experimental design

This study employed a within-subject controlled design comprising nutritional supplement type (five levels: caffeine, nitrate, Ginkgo biloba extract, catechins, and placebo) × mental fatigue state (three levels:initial, fatigued, and post-supplementation). The order of supplement administration was randomized for each participant to minimize order and carryover effects.

All nutritional supplements and the placebo were encapsulated in visually identical capsules. The capsules were coded and randomly allocated by a third party who was not involved in data collection. Both the investigators and participants were blinded to the capsule contents, and the allocation codes were revealed only after all data collection had been completed. This procedure was implemented to minimize the potential influence of expectancy effects, the Hawthorne effect, and other sources of bias on the experimental results [37].

The order of the performance tests was randomized to reduce learning effects. Before each experimental session, the researchers confirmed that participants had completed the required pre-experimental preparations, including abstaining from caffeine and alcohol consumption and avoiding strenuous exercise. Supplement ingestion was supervised on site by the researchers, and any apparent discomfort or adverse reactions occurring during the experiment were documented. All participants completed every experimental condition, with no study discontinuations or serious adverse events.

#### 2.2.1 Experimental equipment

**Figure 1.**
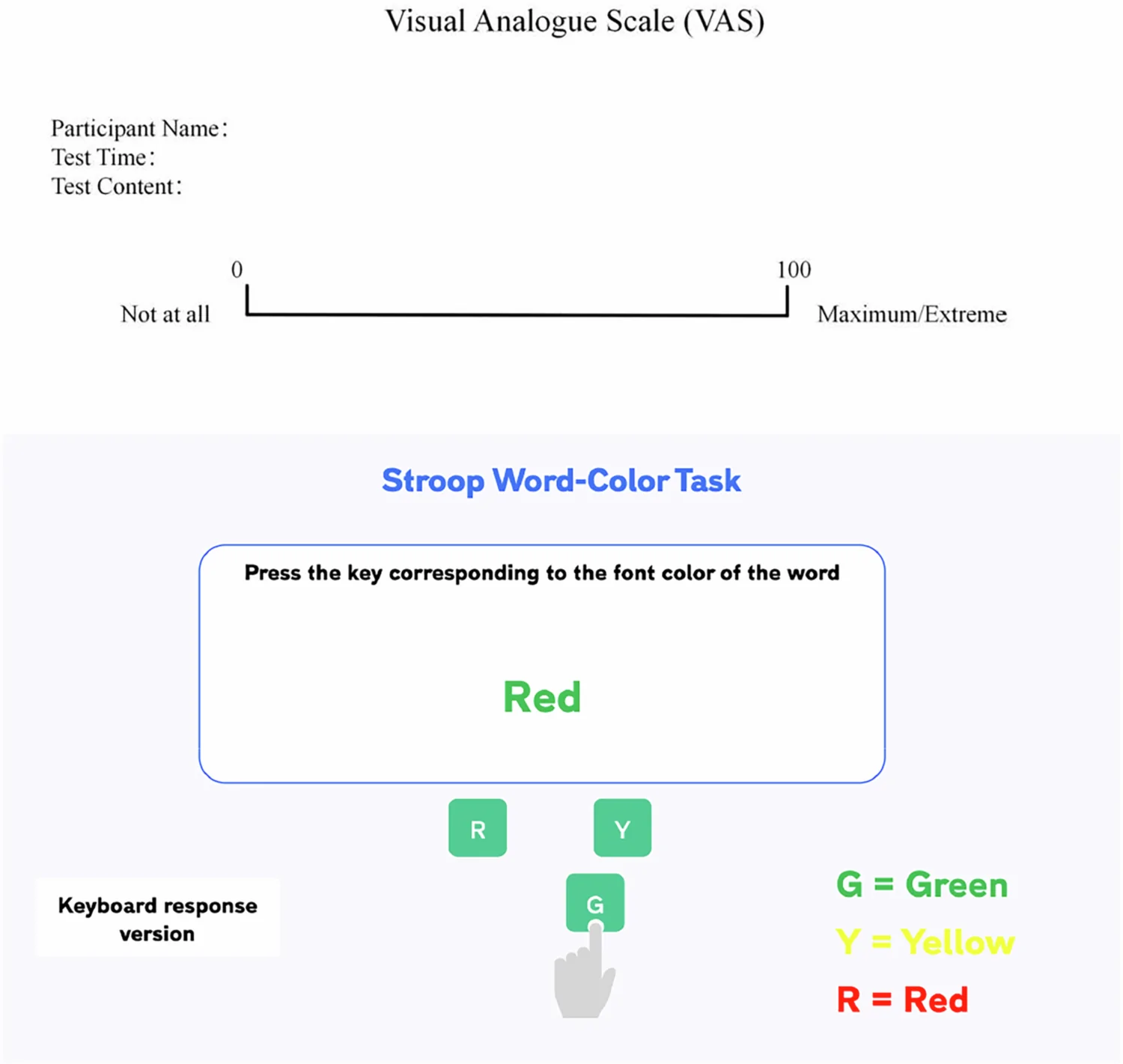
Schematic diagram of VAS and Stroop test.

In this study, a Stroop color–word task was primarily used to induce mental fatigue in the esports athletes, and a Visual Analog Scale (VAS) was used to assess participants' perceived fatigue (Figure 1).

**Figure 2.**
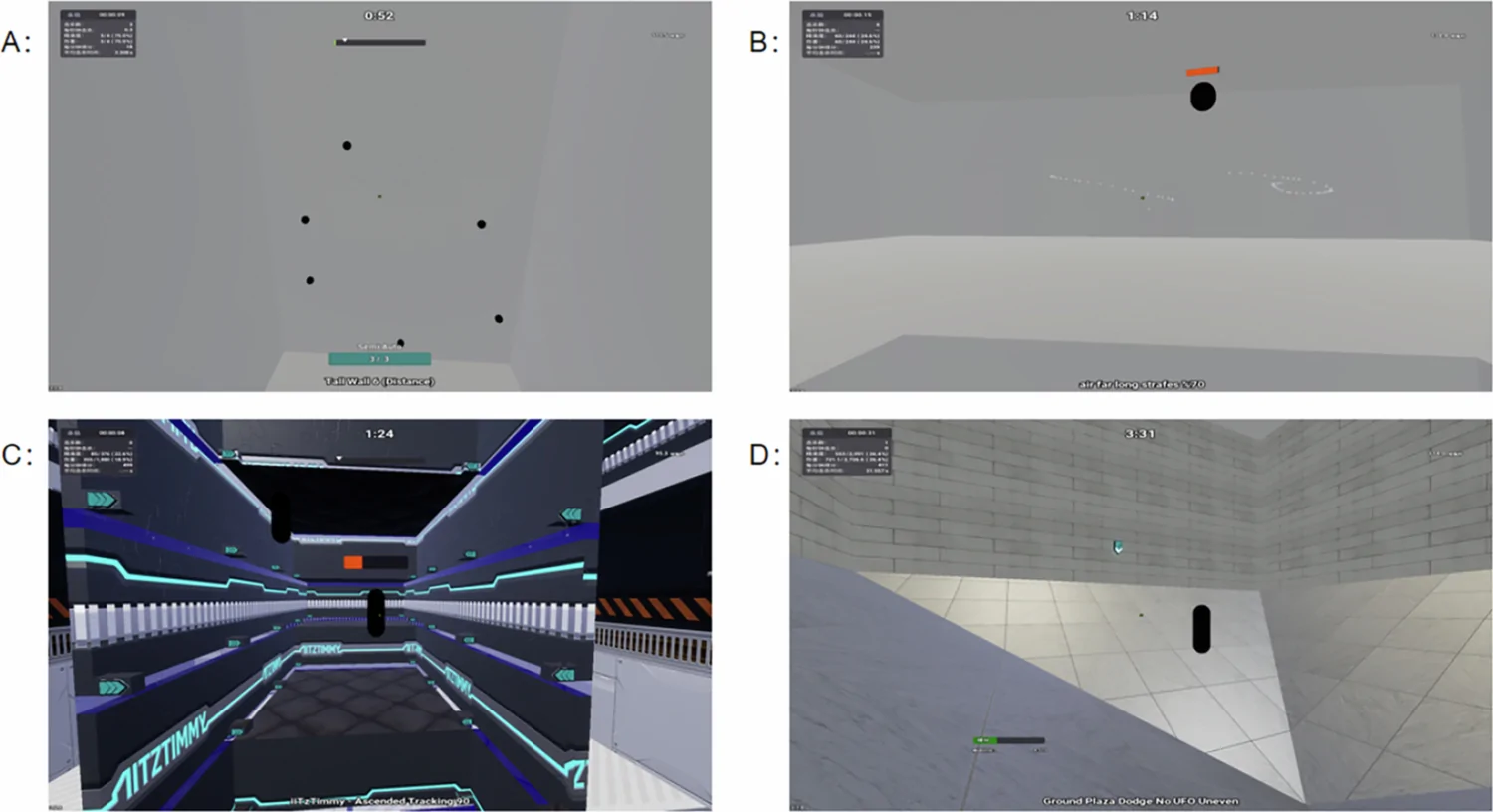
Schematic diagram of test content. Note: A is shooting accuracy test, B is shooting stability test, C is spatial localization test, D is multitasking ability test.

Rogers et al. compared the KovaaK's and Aim Lab aim-training platforms and found that KovaaK's provided a reliable platform for assessing shooting proficiency and could be used in sports research [31]. In the present study, the KovaaK's aim trainer was used to assess athletes' competitive performance following mental fatigue (Figure 2). Shooting accuracy was assessed using the “1 Tall Wall 6 (Distance)” task, which primarily evaluated rapid transitions and clicking between static targets and lasted 60 seconds. Shooting stability was assessed using the “Air far Long Strafes 70%” task, which primarily evaluated the ability to continuously track moving targets and lasted 85 seconds. Spatial localization was assessed using the “IiTzTimmy-Ascended Tracking 90” task, which primarily evaluated the ability to locate and continuously track moving targets in a complex three-dimensional environment and lasted 90 seconds. Multitasking ability was assessed using the “Ground Plaza Dodge No UFO Uneven” task, during which participants continuously tracked and attacked targets while moving and dodging; the task lasted 240 seconds. The four competitive performance abilities were primarily evaluated based on the number of kills, kills per second, accuracy, damage, score per second, and mean time to kill. The final score automatically calculated by the system was used as the final outcome for each test, without further weighting or standardization.

**Figure 3.**
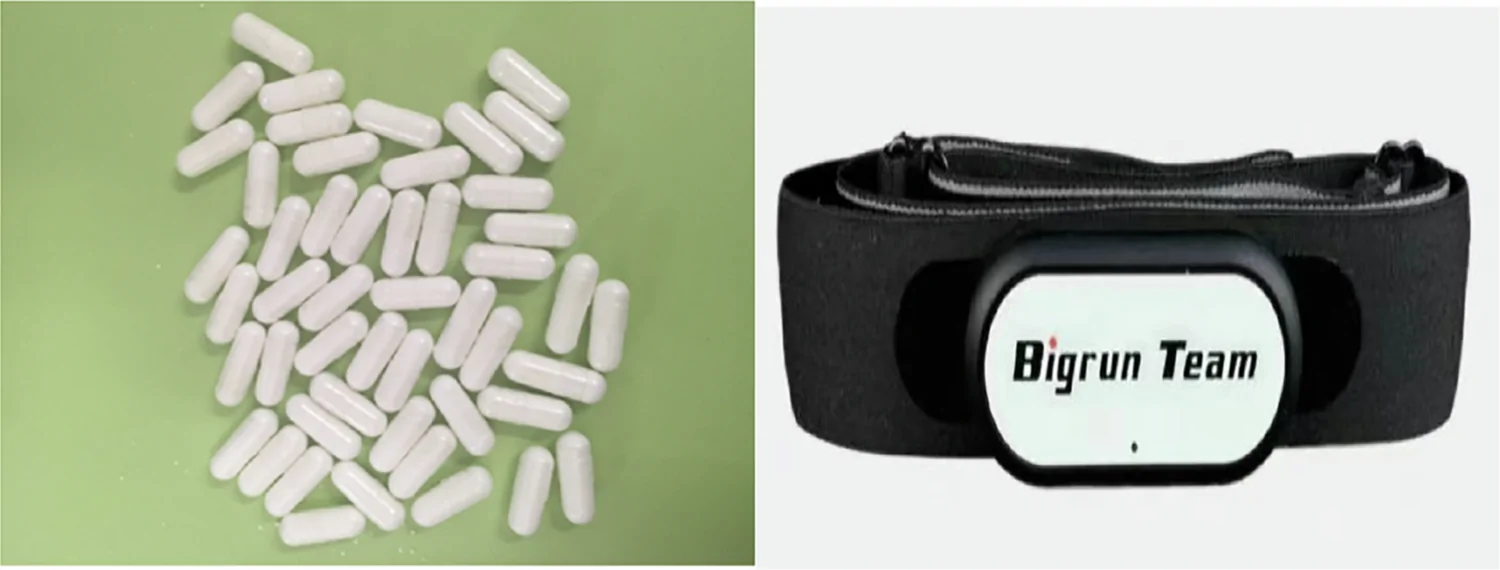
Appearance of the capsules used in the experiment and a schematic illustration of the BigrunTeam chest-strap heart rate sensor.

Participants' HRV was continuously measured using a BigrunTeam chest-strap heart-rate sensor (Figure 3). HRV data were displayed in real time using ECTRI 2.0 and subsequently analyzed using Kubios HRV analysis software to evaluate athletes' autonomic nervous system activity.

#### 2.2.2 Nutritional supplements

Caffeine, nitrate, Ginkgo biloba extract, catechins, and placebo were used as the interventions in this study. All supplements were encapsulated in capsules identical in appearance and packaging to ensure a double-blind experimental design (Figure 3).

The caffeine supplement was MuscleTech Platinum 100% Caffeine, with anhydrous caffeine as its primary active ingredient. In accordance with the International Society of Sports Nutrition (ISSN) position stand on caffeine and exercise performance, an individualized, body-mass-adjusted supplementation protocol was used, with a single caffeine dose of 3 mg/kg [32,38].

The nitrate supplement was derived from iHerb. Based on the nitrate content in beetroot extract (115 mg/g) and the acute nitrate dose commonly used in sports nutrition research (12.8 mmol, equivalent to approximately 794 mg of NO₃^−^), a single nitrate dose of 794 mg was administered [39,40].

The Ginkgo biloba extract supplement was obtained from Vitabiotics Ultra and contained 24% flavonoid glycosides and 6% terpene lactones. In previous studies examining acute cognitive function, a single 600-mg dose of Ginkgo biloba extract has been used to evaluate cognitive performance in healthy participants, with potential effects on attention and psychomotor function being observed [41–43]. Therefore, a single dose of 600 mg was used as the acute Ginkgo biloba extract intervention in the present study.

The catechins supplement was obtained from New Roots Herbal and contained approximately 75% epigallocatechin gallate (EGCG). Based on previous human intervention studies involving green tea extract and EGCG [44], a single 340-mg dose of catechin extract was administered, corresponding to an EGCG intake of approximately 255 mg.

Food-grade starch capsules were used as the placebo.

#### 2.2.3 Experimental procedures

**Figure 4.**
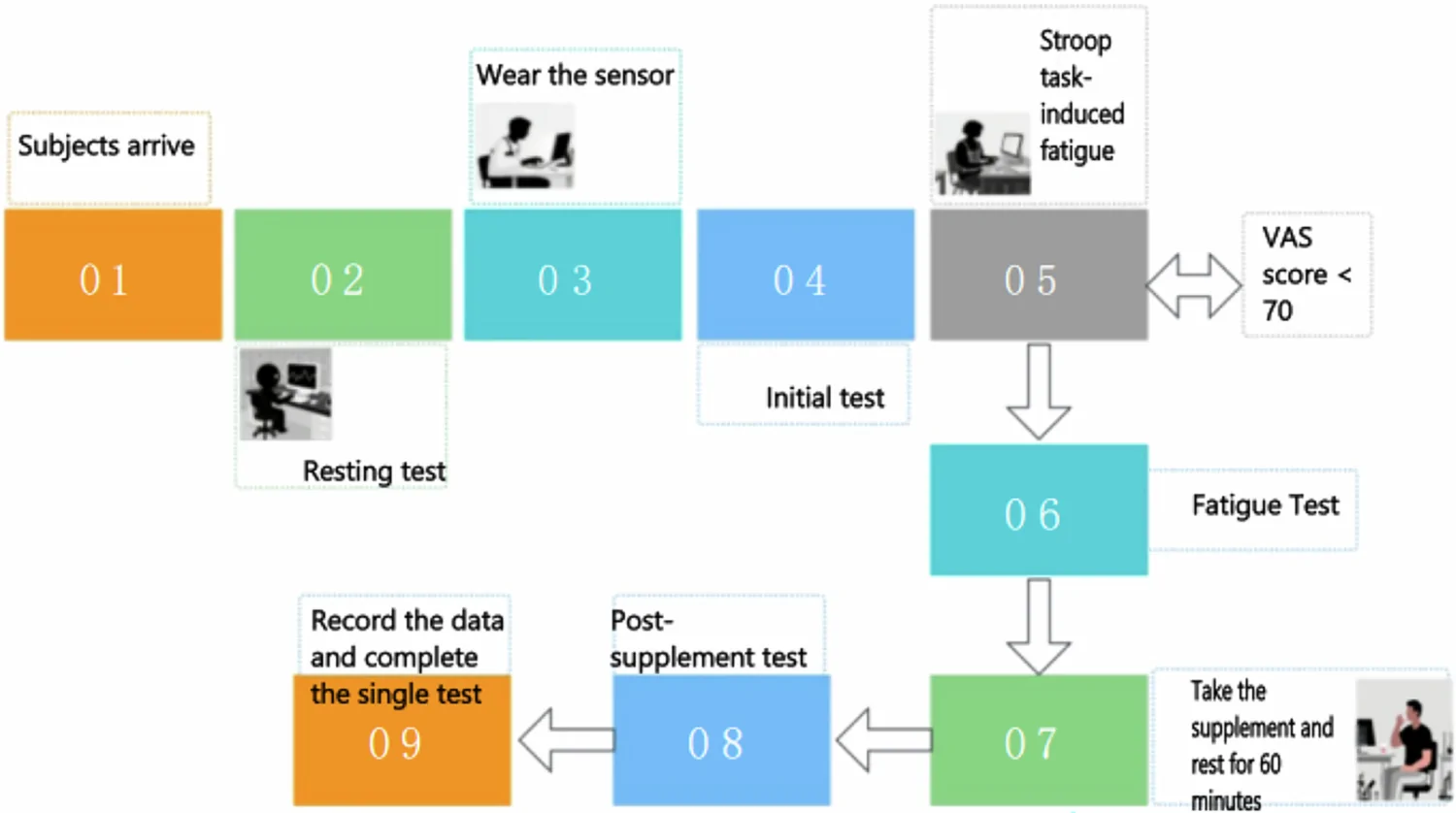
Schematic diagram of the experimental process.

The experimental process is shown in Figure 4. Acclimatization and baseline assessment: Upon arrival at the laboratory, participants remained seated for 10 minutes to acclimatize to the environment. They then wore the heart-rate sensor and remained quietly seated for an additional 10 min, during which baseline HRV data were collected.

Initial performance assessment: Participants completed the four competitive performance tests using the KovaaK's aim trainer, while HRV was recorded concurrently.

Mental-fatigue induction: Participants performed the Stroop task for 30–60 minutes. Perceived fatigue was assessed every 15 minutes using a 100-mm VAS in response to the question, “Do you feel fatigued?” (0 = no fatigue; 100 = extreme fatigue). Given individual differences in susceptibility to mental fatigue [45], the task was terminated when a participant's VAS score reached ≥70, after which the performance tests in the fatigued state were administered immediately [46].

Nutritional supplementation and recovery: After ingesting the assigned nutritional supplement, participants rested for 60 minutes, during which the use of electronic devices was prohibited. They subsequently completed the post-supplementation performance tests, and HRV data were collected.

Repeated experimental sessions: Participants completed the different supplementation conditions on separate experimental days in a randomized order. To reduce potential carryover effects between experimental conditions, a washout period of at least 24 hours was implemented between consecutive sessions based on the half-lives of the supplements: caffeine, 3–7 hours [47,48]; nitrate, 1–2 hours [49]; Ginkgo biloba extract, 4–5 hours [50,51]; and catechins, 1.9–4.6 hours [52,53]. This washout period was intended to reduce the potential acute effects of supplement ingestion and the mental-fatigue task from the preceding session on subsequent testing. The mental-fatigue states assessed in this study were categorized as the initial state, fatigued state, and post-supplementation state.

### 2.3 Outcome measures

#### 2.3.1 Competitive performance measures

Four dimensions of competitive performance were measured: shooting accuracy, shooting stability, spatial localization, and multitasking ability.

Shooting accuracy is a fundamental ability in FPS games. It refers to the action of rapidly moving the crosshair onto an object or enemy and firing a shot to damage or destroy the target [54]. Shooting stability refers to the ability to maintain hand steadiness, operational consistency, and psychological stability [55]. Spatial localization refers to the ability to construct three-dimensional cognition through the integration of multimodal perceptual information [56]. Multitasking ability refers to an individual's cognitive ability to process multiple interrelated subtasks simultaneously in complex scenarios and to switch rapidly between different subtasks in response to environmental changes, thereby maintaining overall task efficiency [57].

#### 2.3.2 Autonomic nervous system measures

In this study, HRV was used to observe changes in participants' autonomic nervous system activity. As an important physiological indicator reflecting the balance between sympathetic and parasympathetic nervous system tone, HRV has been widely used to assess neurophysiological regulation in athletes under mental fatigue [58]. Previous studies have identified the principal HRV indices that are directly physiologically associated with cardiac vagal activity. Accordingly, the following key indices were systematically analyzed in this study.

Heart rate (HR): HR refers to the number of heartbeats per minute and is the most fundamental cardiovascular physiological indicator. It directly reflects the frequency of cardiac contractions. Changes in HR can provide a preliminary indication of how the autonomic nervous system regulates cardiac activity: HR generally increases when the sympathetic nervous system is activated and tends to decrease when the parasympathetic nervous system predominates [59].

R–R interval (RR): The RR interval refers to the time interval between two consecutive R waves on an electrocardiogram and constitutes the basic data used in HRV analysis [58]. Its stability directly reflects the regularity of cardiac rhythm and serves as a fundamental indicator for evaluating the immediate regulation of the heart by the vagus nerve. Autonomic nervous system disturbances induced by mental fatigue are generally first manifested as changes in the magnitude of RR-interval fluctuations.

Root mean square of successive differences (RMSSD): RMSSD is a time-domain index calculated as the root mean square of the differences between successive normal RR intervals. This index is highly sensitive to short-term fluctuations in parasympathetic nervous system activity and is almost unaffected by sympathetic nervous system activity [59]. A higher RMSSD value indicates greater parasympathetic activity and a stronger inhibitory regulatory effect on the heart. RMSSD is also a key indicator of the capacity for rapid autonomic regulation under mental fatigue [58].

Standard deviation of normal-to-normal RR intervals (SDNN): SDNN represents the overall dispersion of all normal RR intervals and reflects the overall functional capacity of the autonomic nervous system to regulate cardiac activity [58]. Unlike RMSSD, which focuses on short-term regulation, SDNN reflects the combined regulatory capacity of the sympathetic and parasympathetic nervous systems. A higher SDNN value indicates a greater overall autonomic regulatory reserve. SDNN can directly reflect autonomic balance over a longer timescale and is also a key indicator for assessing the overall recovery of autonomic nervous system function in esports athletes following mental fatigue [59].

High-frequency power (HF): HF is a frequency-domain HRV index, and changes in HF power specifically reflect the level of parasympathetic nervous system activity [59]. Similar to RMSSD, a higher HF value indicates stronger parasympathetic dominance. HF is a principal frequency-domain indicator for assessing vagal tone and can complement time-domain indices to provide a more comprehensive reflection of parasympathetic nervous system function.

Low-frequency to high-frequency power ratio (LF/HF): LF/HF is a key indicator reflecting the relative balance between the sympathetic and parasympathetic nervous systems. Low-frequency power primarily reflects the combined activity of the sympathetic and parasympathetic nervous systems, whereas HF specifically reflects parasympathetic nervous system activity [58]. Therefore, the LF/HF ratio can directly reflect the relative predominance of sympathetic nervous system activity: an increased ratio indicates sympathetic predominance, whereas a decreased ratio indicates parasympathetic predominance. This index is a key basis for determining whether autonomic balance is disturbed [59].

Overall, RMSSD, RR, and HF were primarily used in this study to accurately reflect changes in parasympathetic nervous system function, whereas SDNN and LF/HF were primarily used to reflect the overall balance between the sympathetic and parasympathetic nervous systems [58].

### 2.4 Data analysis

Statistical analyzes were performed using SPSS Statistics. The normality of all continuous variables was first assessed using the Shapiro–Wilk test. As all variables satisfied the assumption of normality, parametric tests were used for all subsequent analyzes. A two-way repeated-measures analysis of variance (ANOVA) was conducted, with supplement type and mental fatigue state included as within-subject factors, to examine their main and interaction effects on competitive performance measures. When the repeated-measures ANOVA indicated a significant main or interaction effect, post hoc pairwise comparisons were performed with Bonferroni correction to control the family-wise error rate (FWER) arising from multiple comparisons. The assumption of sphericity for the repeated-measures factors was evaluated using Mauchly's test. The F statistic, *p* value, and partial eta squared (ηp^2^) were reported, with ηp^2^ used as the measure of effect size. According to Cohen's effect-size criteria, ηp^2^ values of approximately 0.01, 0.06, and 0.14 represent small, medium, and large effects, respectively, and were used to evaluate the practical magnitude of the observed effects beyond statistical significance [60]. All continuous variables are presented as the mean ± standard deviation. All statistical tests were two-tailed, with statistical significance set at *p* < 0.05.

## 3 Results

### 3.1 Effects of different nutritional supplements on the recovery of shooting accuracy in esports athletes following mental fatigue

**Figure 5.**
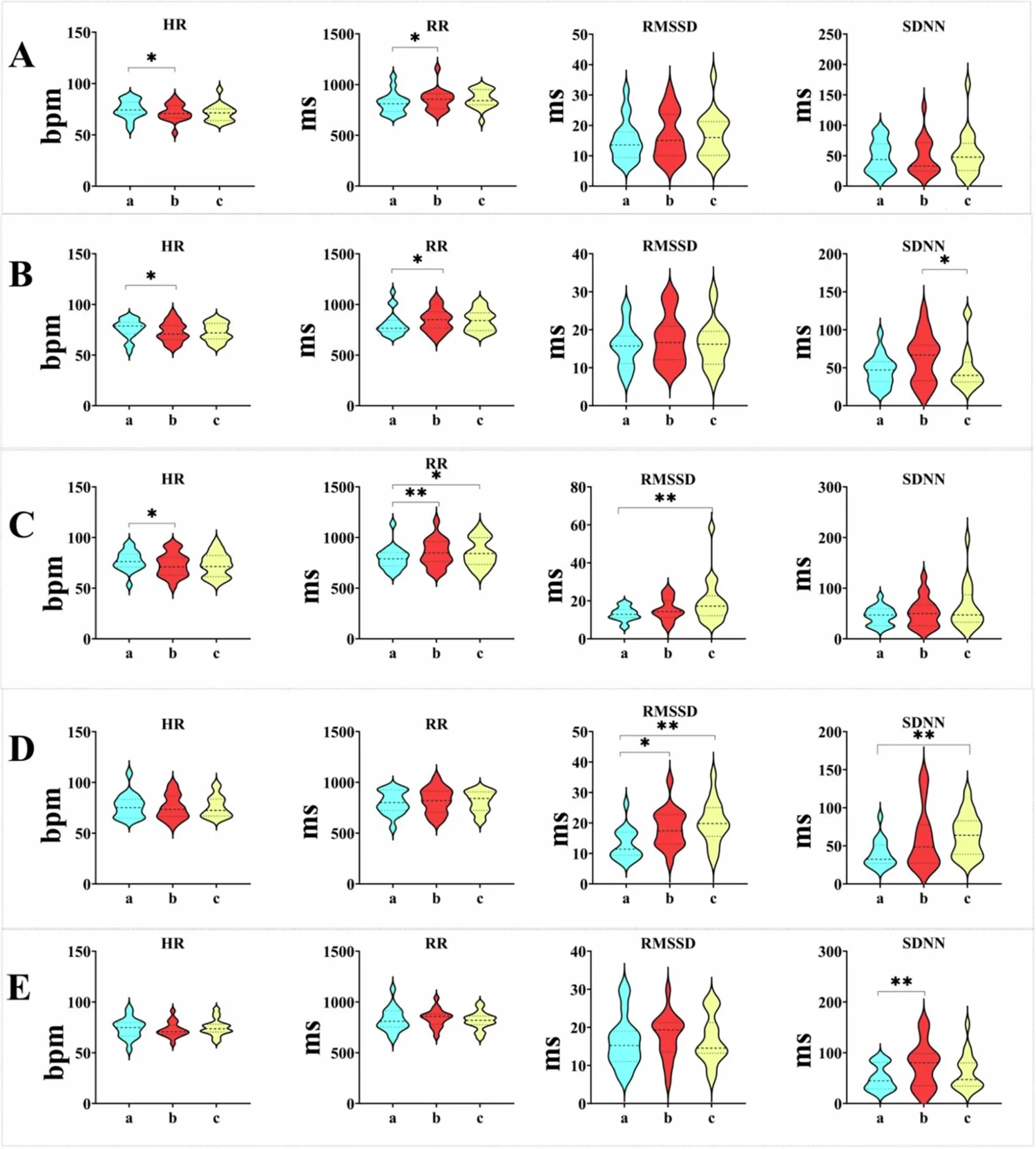
HRV outcomes associated with shooting accuracy across mental fatigue states under nutritional supplementation in esports athletes. Note: a, b, and c denote the Initial State, Fatigued State, and Post-Supplementation State, respectively; A, B, C, and D represent the nutritional supplements including caffeine, nitrate, Ginkgo biloba extract, and Catechins supplement, respectively; E is the placebo. * indicates *p* < 0.05, ** indicates *p* < 0.01, and *** indicates *p* < 0.001.

Descriptive statistics for the shooting accuracy scores of esports athletes under the different supplementation conditions are presented in Table 2. The two-way repeated-measures ANOVA showed that the main effect of supplement type was not significant (F = 2.000, *p* = 0.103, ηp^2^ = 0.095). The main effect of mental fatigue state was significant (F = 60.376, *p* = 0.001, ηp^2^ = 0.761), with a large effect size. The interaction between supplement type and mental fatigue state was also significant (F = 5.531, *p* = 0.001, ηp^2^ = 0.225) and represented a large effect (Table 3). Pairwise comparisons showed that, relative to the fatigued state, shooting accuracy scores increased significantly following the ingestion of caffeine, nitrate, Ginkgo biloba extract, and catechins (*p* = 0.001, *p* = 0.001, *p* = 0.013, and *p* = 0.001, respectively). No significant recovery effect was observed under the placebo condition (*p* = 1.000) (Table 4). Further Bonferroni-adjusted pairwise comparisons among the supplements that produced significant recovery effects showed no significant differences between caffeine, nitrate, Ginkgo biloba extract, and catechins in the post-supplementation state (all *p* > 0.05) (Table 5). The HRV results (Figure 5) showed significant changes in RMSSD under the Ginkgo biloba extract and catechin conditions, whereas significant changes in SDNN were observed under the nitrate and catechin conditions.

**Table 2.** Descriptive statistics of shooting accuracy scores under different nutritional supplements (M ± SD).

Nutritional supplement type	Initial state	Fatigued state	Post-supplementation state
Caffeine	69.40 ± 7.24	66.60 ± 9.15	71.50 ± 7.65
Nitrate	71.95 ± 5.31	67.80 ± 6.63	72.30 ± 4.79
Ginkgo biloba extract	72.60 ± 8.70	69.20 ± 9.86	71.40 ± 8.90
Catechins	71.10 ± 7.59	66.90 ± 6.90	69.40 ± 6.86
Placebo	69.10 ± 8.18	66.85 ± 8.06	67.40 ± 8.37

**Table 3.** Results of the two-way repeated-measures ANOVA for shooting accuracy scores.

	F	*p*	$\eta_{p}^{2}$
Nutritional supplement type	2.000	0.103	0.095
Mental fatigue state	60.376	0.001	0.761
Interaction effect	5.531	0.001	0.225

**Table 4.** Pairwise comparisons of shooting accuracy across mental fatigue states under each supplementation condition in esports athletes.

					95% CI	
Supplement type	Mental fatigue state		MD ± SE	*p*	LB	UB
Caffeine	Initial Initial Fatigued	Fatigued Post-supplementation Post-supplementation	2.80 ± 0.87 −2.10 ± 0.51 −4.90 ± 0.83	0.013 0.002 0.001	0.526 −3.432 −7.087	5.074 −0.768 −2.713
Nitrate	Initial Initial Fatigued	Fatigued Post-supplementation Post-supplementation	4.15 ± 0.80 −0.35 ± 5.30 −4.50 ± 0.84	0.001 1.000 0.001	2.044 −1.740 −6.717	6.256 1.040 −2.283
Ginkgo biloba extract	Initial Initial Fatigued	Fatigued Post-supplementation Post-supplementation	3.40 ± 0.81 1.20 ± 0.64 −2.20 ± 0.68	0.002 0.222 0.013	1.267 −0.467 −3.993	5.533 2.867 −0.407
Catechins	Initial Initial Fatigued	Fatigued Post-supplementation Post-supplementation	4.20 ± 0.42 1.70 ± 0.41 −2.50 ± 0.36	0.001 0.002 0.001	3.096 0.621 −3.443	5.304 2.779 −1.557
Placebo	Initial Initial Fatigued	Fatigued Post-supplementation Post-supplementation	2.25 ± 0.46 1.70 ± 0.65 −0.55 ± 0.68	0.001 0.053 1.000	1.032 −0.015 −2.321	3.468 3.415 1.221
Overall	Initial Initial Fatigued	Fatigued Post-supplementation Post-supplementation	3.36 ± 0.38 0.43 ± 0.29 −2.93 ± 0.32	0.001 0.463 0.001	2.353 −0.331 −3.764	4.367 1.191 −2.096

**Table 5.** Pairwise comparisons among supplementation conditions for shooting accuracy within each mental fatigue state in esports athletes.

Mental Fatigue State	Supplement type		MD ± SE	*p*	95% CI	
					LB	UB
Initial State	Caffeine Caffeine Caffeine Nitrate Nitrate Ginkgo biloba extract	Nitrate Ginkgo biloba extract Catechins Ginkgo biloba extract Catechins Catechins	−2.55 ± 1.49 −3.20 ± 1.23 −1.70 ± 1.49 −0.65 ± 1.47 0.85 ± 1.08 1.50 ± 1.58	1.000 0.172 1.000 1.000 1.000 1.000	−7.271 −7.091 −6.442 −5.308 −2.583 −3.521	2.171 0.691 3.042 4.008 4.283 6.521
Fatigued State	Caffeine Caffeine Caffeine Nitrate Nitrate Ginkgo biloba extract	Nitrate Ginkgo biloba extract Catechins Ginkgo biloba extract Catechins Catechins	−1.20 ± 1.98 −2.60 ± 1.34 −0.30 ± 1.62 −1.40 ± 1.78 0.90 ± 1.47 2.30 ± 1.88	1.000 0.667 1.000 1.000 1.000 1.000	−7.470 −6.842 −5.444 −7.042 −3.767 −3.678	5.070 1.642 4.844 4.242 5.567 8.278
Post-Supplementation State	Caffeine Caffeine Caffeine Nitrate Nitrate Ginkgo biloba extract	Nitrate Ginkgo biloba extract Catechins Ginkgo biloba extract Catechins Catechins	−0.80 ± 1.54 −0.10 ± 1.36 2.10 ± 1.38 0.90 ± 1.39 2.90 ± 1.14 2.00 ± 1.49	1.000 1.000 1.000 1.000 0.200 1.000	−5.688 −4.201 −2.280 −3.516 −0.725 −2.735	4.088 4.401 6.480 5.316 6.525 6.735

### 3.2 Effects of different nutritional supplements on the recovery of shooting stability in esports athletes following mental fatigue

Descriptive statistics for the shooting stability scores of esports athletes under the different supplementation conditions are presented in Table 6. The two-way repeated-measures ANOVA showed a significant main effect of supplement type (F = 4.859, *p* = 0.002, ηp^2^ = 0.204), with a large effect size. The main effect of mental fatigue state was also significant (F = 68.045, *p* = 0.001, ηp^2^ = 0.782) and exhibited the largest effect size. A significant interaction between supplement type and mental fatigue state was observed (F = 3.028, *p* = 0.003, ηp^2^ = 0.137), with a moderate-to-large effect size (Table 7).

**Table 6.** Descriptive statistics of shooting stability scores under different nutritional supplements (M ± SD).

Nutritional supplement type	Initial state	Fatigued state	Post-supplementation state
Caffeine	3412.40 ± 465.11	3135.70 ± 491.17	3536.10 ± 452.46
Nitrate	3443.60 ± 528.87	3216.45 ± 527.50	3440.30 ± 512.37
Ginkgo biloba extract	3664.90 ± 455.01	3556.50 ± 425.72	3641.95 ± 398.98
Catechins	3573.70 ± 475.53	3342.90 ± 558.79	3514.70 ± 477.10
Placebo	3601.50 ± 414.00	3370.85 ± 431.28	3554.40 ± 467.91

**Table 7.** Results of the two-way repeated-measures ANOVA for shooting stability scores.

	F	*p*	$\eta_{p}^{2}$
Nutritional supplement type	4.859	0.002	0.204
Mental fatigue state	68.045	0.001	0.782
Interaction effect	3.028	0.003	0.137

**Figure 6.**
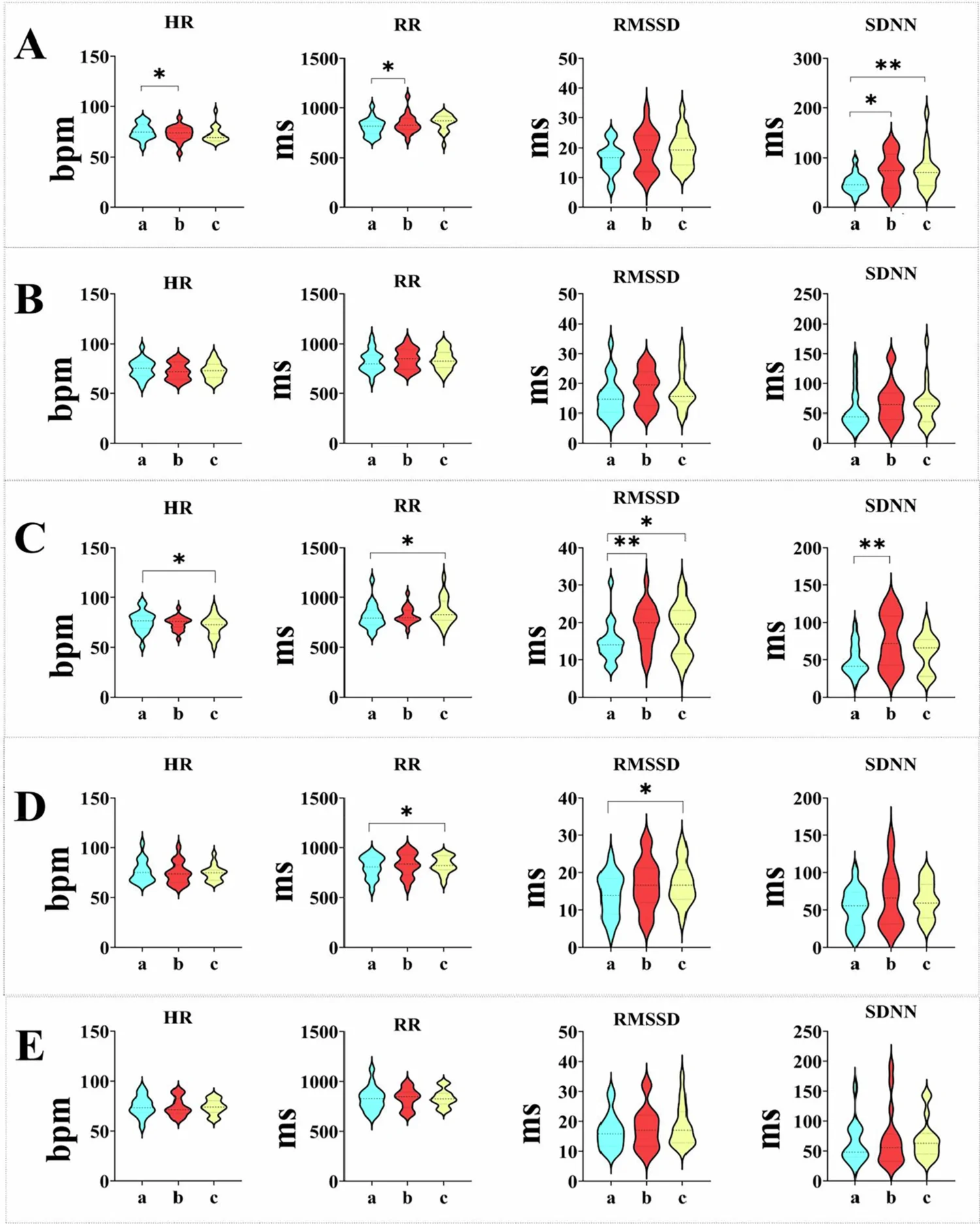
HRV outcomes associated with shooting stability across mental fatigue states under nutritional supplementation in esports athletes. Note: a, b, and c denote the Initial State, Fatigued State, and Post-Supplementation State, respectively; A, B, C, and D represent the nutritional supplements including caffeine, nitrate, Ginkgo biloba extract, and Catechins supplement, respectively; E is the placebo. * indicates *p* < 0.05, ** indicates *p* < 0.01, and *** indicates *p* < 0.001.

Pairwise comparisons showed that, relative to the fatigued state, shooting stability scores increased significantly following the ingestion of caffeine, nitrate, and catechins (*p* = 0.001, *p* = 0.001, and *p* = 0.009, respectively). Under the placebo condition, shooting stability scores showed a marginal trend toward recovery, but the difference did not reach statistical significance (*p* = 0.053). Ginkgo biloba extract did not produce a significant recovery effect (*p* = 0.061) (Table 8). Further Bonferroni-adjusted pairwise comparisons among the supplements that produced significant recovery effects showed no significant differences between caffeine, nitrate, and catechins in the post-supplementation state (all *p* > 0.05) (Table 9). As shown in Figure 6, the HRV results indicated significant changes in RMSSD under the Ginkgo biloba extract and catechin conditions, whereas significant changes in SDNN were observed under the caffeine and Ginkgo biloba extract conditions.

**Table 8.** Pairwise comparisons of shooting stability across mental fatigue states under each supplementation condition in esports athletes.

					95% CI	
Supplement type	Mental fatigue state		MD ± SE	*p*	LB	UB
Caffeine	Initial Initial Fatigued	Fatigued Post-supplementation Post-supplementation	276.70 ± 54.96 −123.70 ± 64.11 −400.40 ± 65.88	0.001 0.206 0.001	132.437 −291.992 −573.347	420.963 −44.592 −227.453
Nitrate	Initial Initial Fatigued	Fatigued Post-supplementation Post-supplementation	227.15 ± 45.03 3.30 ± 42.46 −223.85 ± 38.37	0.001 1.000 0.001	108.931 −108.161 −324.580	345.369 114.761 −123.120
Ginkgo biloba extract	Initial Initial Fatigued	Fatigued Post-supplementation Post-supplementation	108.40 ± 39.61 22.95 ± 44.96 −85.45 ± 33.80	0.039 1.000 0.061	4.419 −95.068 −174.168	212.381 140.968 3.268
Catechins	Initial Initial Fatigued	Fatigued Post-supplementation Post-supplementation	230.80 ± 32.72 59.00 ± 36.18 −171.80 ± 50.83	0.001 0.358 0.009	144.898 −35.966 −305.239	316.702 153.966 −38.361
Placebo	Initial Initial Fatigued	Fatigued Post-supplementation Post-supplementation	230.65 ± 27.70 47.10 ± 68.54 −183.55 ± 70.54	0.001 1.000 0.053	157.948 −132.836 −368.734	303.352 227.036 1.634
Overall	Initial Initial Fatigued	Fatigued Post-supplementation Post-supplementation	214.74 ± 20.40 1.73 ± 22.66 −213.01 ± 20.37	0.001 1.000 0.001	161.188 −57.749 −266.488	268.292 61.209 −159.532

**Table 9.** Pairwise comparisons among supplementation conditions for shooting stability within each mental fatigue state in esports athletes.

Mental fatigue state	Supplement type		MD ± SE	*p*	95% CI	
					LB	UB
Initial state	Caffeine Caffeine Nitrate	Nitrate Catechins Catechins	−31.20 ± 68.85 −161.30 ± 85.47 −130.10 ± 65.60	1.000 0.745 0.620	−249.707 −432.570 −338.288	187.307 109.970 78.088
Fatigued state	Caffeine Caffeine Nitrate	Nitrate Catechins Catechins	−80.75 ± 74.76 −207.20 ± 100.70 −126.45 ± 81.61	1.000 0.536 1.000	−318.017 −526.792 −385.455	156.517 112.392 132.555
Post-supplementation state	Caffeine Caffeine Nitrate	Nitrate Catechins Catechins	95.80 ± 79.92 21.40 ± 54.87 −74.40 ± 79.86	1.000 1.000 1.000	−157.834 −152.729 −327.867	349.434 195.529 179.067

### 3.3 Effects of different nutritional supplements on the recovery of spatial localization in esports athletes following mental fatigue

**Figure 7.**
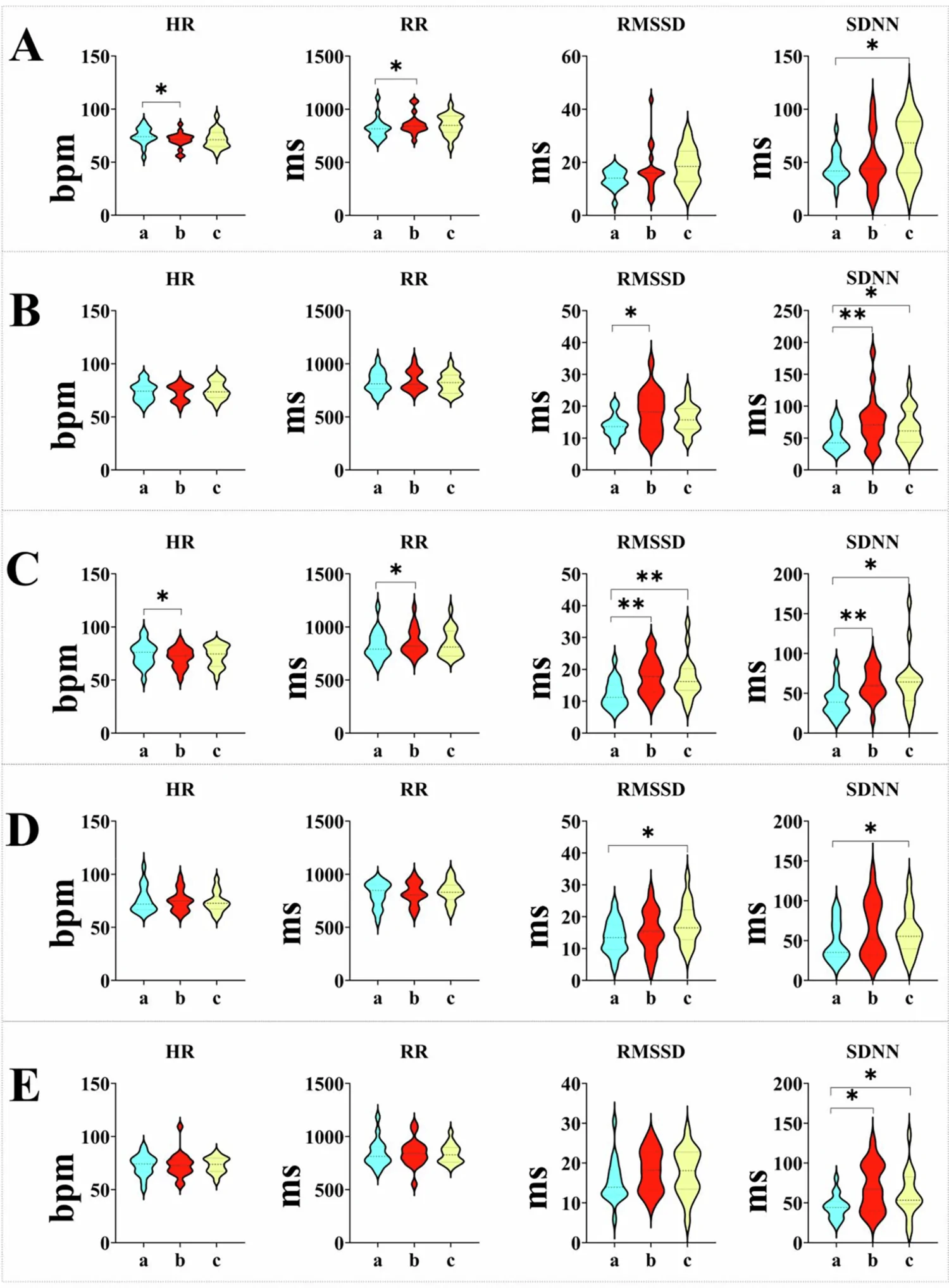
HRV outcomes associated with spatial localization across mental fatigue states under nutritional supplementation in esports athletes. Note: a, b, and c denote the Initial State, Fatigued State, and Post-Supplementation State, respectively; A, B, C, and D represent the nutritional supplements including caffeine, nitrate, Ginkgo biloba extract, and Catechins supplement, respectively; E is the placebo. * indicates *p* < 0.05, ** indicates *p* < 0.01, and *** indicates *p* < 0.001.

Descriptive statistics for the spatial localization scores of esports athletes under the different supplementation conditions are presented in Table 10. The two-way repeated-measures ANOVA showed that the main effect of supplement type was not significant (F = 2.160, *p* = 0.082, ηp^2^ = 0.102), whereas the main effect of mental fatigue state was significant (F = 49.413, *p* = 0.001, ηp^2^ = 0.722) and exhibited the largest effect size. The interaction between supplement type and mental fatigue state was not significant (F = 1.567, *p* = 0.139, ηp^2^ = 0.076) (Table 11). Pairwise comparisons showed that spatial localization scores in the initial state were significantly higher than those in the fatigued state (*p* = 0.001), but did not differ significantly from those in the post-supplementation state (*p* = 0.273). Spatial localization scores in the post-supplementation state were significantly higher than those in the fatigued state (*p* = 0.001) (Table 12). The HRV analysis (Figure 7) showed significant changes in RMSSD under the nitrate, Ginkgo biloba extract, and catechin conditions. Significant changes in SDNN were observed under all four active supplementation conditions.

**Table 10.** Descriptive statistics of spatial localization scores under different nutritional supplements (M ± SD).

Nutritional supplement type	Initial state	Fatigued state	Post-supplementation state
Caffeine	2829.23 ± 1014.76	2529.86 ± 885.36	3002.68 ± 961.37
Nitrate	3027.05 ± 1105.00	2727.48 ± 900.19	3226.64 ± 882.19
Ginkgo biloba extract	3290.28 ± 1110.80	2850.27 ± 990.42	3324.51 ± 967.88
Catechins	3101.72 ± 1195.98	2775.38 ± 1040.59	3263.98 ± 1104.45
Placebo	3412.69 ± 1329.60	3036.04 ± 1341.00	3296.90 ± 1416.11

**Table 11.** Results of the two-way repeated-measures ANOVA for spatial localization scores.

	F	*p*	$\eta_{p}^{2}$
Nutritional supplement type	2.160	0.082	0.102
Mental fatigue state	49.413	0.001	0.722
Interaction effect	1.567	0.139	0.076

**Table 12.** Results of pairwise comparisons on spatial localization of esports athletes under different mental fatigue state.

Mental fatigue state		MD ± SE	*p*	95% CI	
				LB	UB
Initial	Fatigued	348.39 ± 47.05	0.001	224.883	471.895
Initial	Post-supplementation	−90.75 ± 50.99	0.273	−224.599	43.101
Fatigued	Post-supplementation	−439.14 ± 41.38	0.001	−547.765	−330.511

### 3.4 Effects of different nutritional supplements on the recovery of multitasking ability in esports athletes following mental fatigue

**Figure 8.**
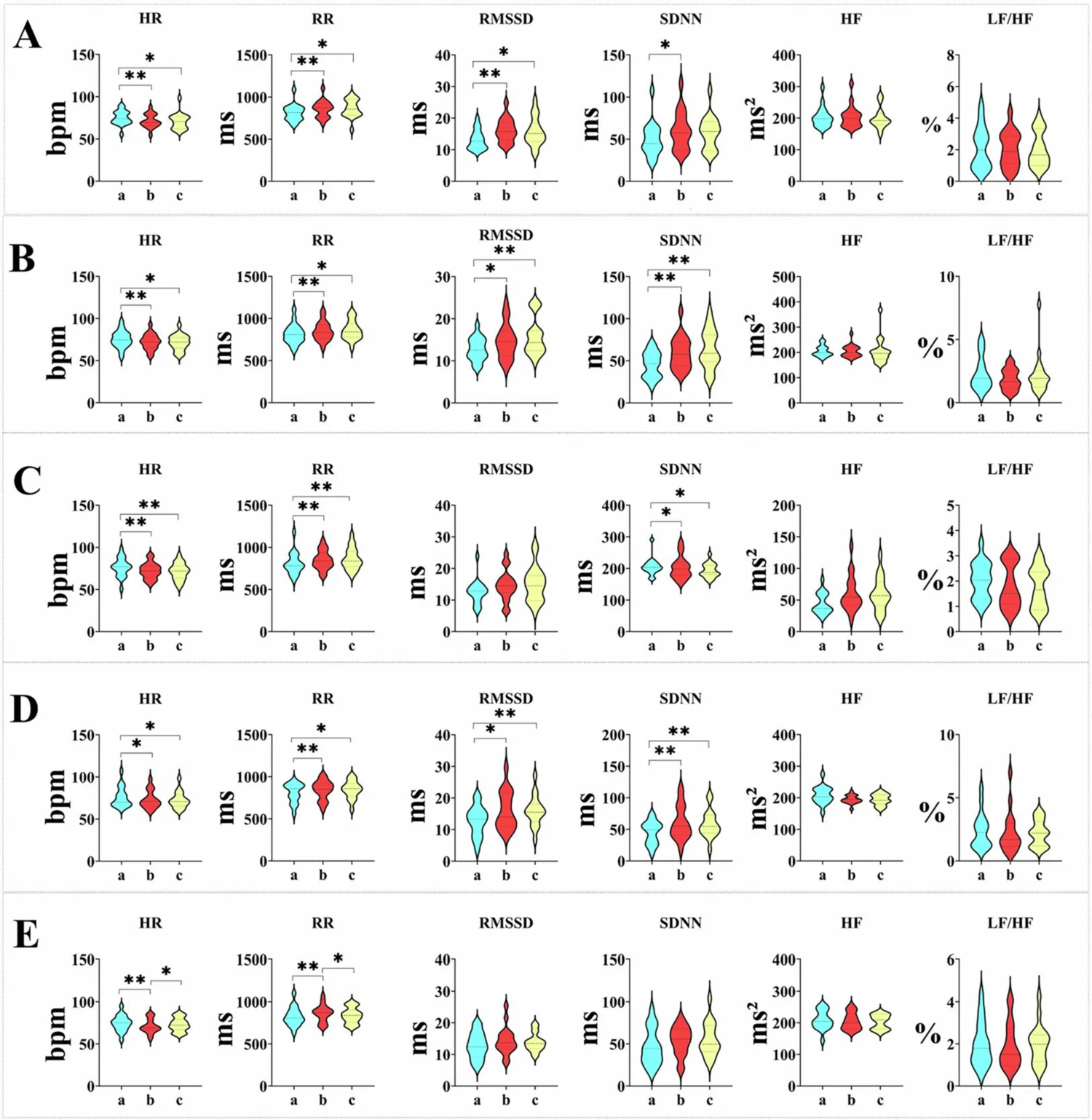
HRV outcomes associated with multitasking ability across different mental fatigue state under nutritional supplementation in esports athletes. Note: a, b, and c denote the Initial State, Fatigued State, and Post-Supplementation State, respectively; A, B, C, and D represent the nutritional supplements including caffeine, nitrate, Ginkgo biloba extract, and Catechins supplement, respectively; E is the placebo. * indicates *p* < 0.05, ** indicates *p* < 0.01, and *** indicates *p* < 0.001.

Descriptive statistics for the multitasking ability scores of esports athletes under the different supplementation conditions are presented in Table 13. The two-way repeated-measures ANOVA showed that the main effect of supplement type was not significant (F = 1.657, *p* = 0.169, ηp^2^ = 0.080). The main effect of mental fatigue state was significant (F = 25.078, *p* = 0.001, ηp^2^ = 0.569) and exhibiting the largest effect size. However, the interaction between these two factors was not significant (F = 1.809, *p* = 0.079, ηp^2^ = 0.087) (Table 14). Pairwise comparisons showed that multitasking ability scores in the initial state were significantly higher than those in the fatigued state (*p* = 0.001) but significantly lower than those in the post-supplementation state (*p* = 0.001). Multitasking ability scores in the post-supplementation state were significantly higher than those in the fatigued state (*p* = 0.001) (Table 15). The HRV results showed no significant changes in HF or LF/HF under any supplementation condition, whereas significant changes were observed in RMSSD and SDNN (Figure 8).

**Table 13.** Descriptive statistics of multitasking ability performance scores under different nutritional supplements (M ± SD).

Nutritional supplement type	Initial state	Fatigued state	Post-supplementation state
Caffeine	64.53 ± 4.15	56.41 ± 4.84	70.44 ± 3.91
Nitrate	71.80 ± 5.01	68.45 ± 5.26	75.10 ± 4.52
Ginkgo biloba extract	71.69 ± 5.10	71.14 ± 5.21	76.03 ± 5.12
Catechins	65.39 ± 5.00	65.77 ± 4.76	73.00 ± 4.60
Placebo	66.24 ± 5.57	68.00 ± 5.79	78.24 ± 5.55

**Table 14.** Results of the two-factor repeated-measures ANOVA for multitasking ability scores.

	F	*p*	$\eta_{p}^{2}$
Nutritional supplement type	1.657	0.169	0.080
Mental fatigue state	25.078	0.001	0.569
Interaction effect	1.809	0.079	0.087

**Table 15.** Results of pairwise comparisons on multitasking ability of esports athletes under different mental fatigue states.

Mental fatigue state		MD ± SE	*p*	95% CI	
				LB	UB
Initial	Fatigued	4.16 ± 1.31	0.001	1.703	6.608
Initial	Post-supplementation	−5.23 ± 1.06	0.001	−7.771	−2.697
Fatigued	Post-supplementation	−9.39 ± 1.46	0.001	−12.438	−6.341

## 4 Discussion

### 4.1 Effects of different nutritional supplements on shooting accuracy in esports athletes

Previous studies have indicated that nitrate, through its reduction to nitric oxide in vivo, can improve microcirculation and cerebral perfusion and may maintain or enhance the fine motor control associated with executive function under cognitive or physical load. These findings suggest that nitrate may benefit performance in tasks requiring highly precise visuomotor coordination [16,17]. Caffeine, a well-established central nervous system stimulant, has been widely demonstrated to increase arousal, accelerate information processing, and sustain attention [9,10,13,32,35]. Several studies have also reported that Ginkgo biloba extract and catechins may improve attention, information-processing speed, and fatigue-related outcomes and, under certain conditions, influence cognitive performance through vascular, antioxidant, or neuroprotective pathways [20,25,26].

The present results showed that, compared with the fatigued state, all four supplements produced significant recovery effects on shooting accuracy (*p* = 0.001, *p* = 0.001, *p* = 0.013, and *p* = 0.001 for caffeine, nitrate, Ginkgo biloba extract, and catechins, respectively). No significant differences in recovery effects were observed among the four supplements. Regarding HRV, significant changes in RMSSD were observed under the Ginkgo biloba extract and catechin conditions, whereas significant changes in SDNN were observed under the nitrate and catechin conditions. Caffeine did not produce consistent significant changes in the principal HRV indices.

The improvement in shooting accuracy without marked changes in HRV under the caffeine condition may be more closely related to increased arousal, improved allocation of attentional resources, and central nervous system regulation, rather than short-term changes in autonomic indices [9,13,32]. The recovery observed under the nitrate condition is consistent with previous findings suggesting that nitrate may improve cerebral perfusion and cognitive performance through nitric oxide-related pathways [17,39]. Under the Ginkgo biloba extract and catechin conditions, both competitive performance recovery and changes in some HRV indices were observed, suggesting that autonomic regulation may be involved in the recovery process. However, it remains unclear whether the observed HRV changes directly contributed to the improvements in behavioral performance. Overall, these findings support the possibility that acute nutritional supplementation may facilitate the recovery of shooting accuracy in esports athletes following mental fatigue. Nevertheless, the specific underlying mechanisms require further investigation using additional neurophysiological measures.

### 4.2 Effects of different nutritional supplements on shooting stability in esports athletes

Caffeine has been shown to improve movement stability and sustained control performance by enhancing alertness and the consistency of motor control [13,14]. Several clinical and experimental studies have demonstrated that catechins can enhance sustained attention and working-memory efficiency, thereby potentially supporting the maintenance of movement consistency [25,26]. Owing to its effects on blood flow and its potential cortisol-inhibitory action, Ginkgo biloba extract has been proposed to alleviate stress-related fluctuations in motor performance [20–22]. In addition, several studies have reported associations between HRV indices, including RMSSD and SDNN, and movement stability or consistency, suggesting that autonomic recovery may represent one of the physiological bases underlying the recovery of movement stability [59,61].

The present study showed that, compared with the fatigued state, caffeine, nitrate, and catechins significantly improved shooting stability following fatigue (*p* = 0.001, *p* = 0.001, and *p* = 0.009, respectively), with no significant differences among the effects of the three supplements. Among the HRV indices, RMSSD changed significantly under the Ginkgo biloba extract and catechin conditions, whereas SDNN changed significantly under the caffeine and Ginkgo biloba extract conditions. Ginkgo biloba extract did not significantly improve shooting stability, possibly because the magnitude of its effect following a single administration was relatively small; its beneficial effects on motor control may generally require regular, long-term intake to become apparent [21,22]. Under the placebo condition, shooting stability scores also tended to increase after the 60-minutes recovery period, with the difference approaching statistical significance (*p* = 0.053).

The improvement in shooting stability under the caffeine condition, accompanied by an improvement in SDNN, may be attributable to caffeine's ability both to enhance central alertness and to exert short-term effects on the overall cardiac autonomic regulatory reserve, thereby facilitating the recovery of movement consistency [13,32]. The concurrent increase in RMSSD and improvement in shooting stability under the catechin condition suggest that catechins may promote shooting stability by enhancing both parasympathetic recovery and sustained attention [25,26]. Although nitrate improved shooting stability, no consistent changes in HRV were observed. Its effects may therefore be mediated primarily through peripheral blood flow, muscular stability, and metabolic support rather than through short-term autonomic regulation [17]. Although Ginkgo biloba extract did not significantly improve shooting stability in the present study, directional changes in performance accompanied by changes in HRV signals suggest that its effects may be relatively small or may require a longer intervention period to become significant. The marginal recovery trend observed under the placebo condition indicates that improvements in shooting stability following mental fatigue may not be attributable solely to nutritional supplementation but may also be influenced by factors such as natural recovery and quiet rest.

### 4.3 Effects of different nutritional supplements on spatial localization in esports athletes

Mental fatigue can reduce the efficiency of perceptual integration and increase spatial errors [8,56]. Different supplements may affect spatial localization through distinct mechanisms. For example, nitrate may support perceptual processing by increasing cerebral blood flow [17]; caffeine may enhance visual-processing speed and attentional allocation [10]; Ginkgo biloba extract may help reduce interference and improve concentration [21]; and catechins may enhance spatial working-memory efficiency [25,26]. In addition, autonomic nervous system balance may enhance the excitability of the perceptual cortex, thereby improving spatial localization performance [61,62].

The present study showed that spatial localization scores after the 60-minutes recovery period were significantly higher than those in the fatigued state. However, none of the four supplements demonstrated a significant recovery effect on spatial localization. Regarding HRV, significant changes in RMSSD were observed under the nitrate, Ginkgo biloba extract, and catechin conditions, whereas significant changes in SDNN were observed under all four active supplementation conditions.

These findings may be explained by the fact that spatial localization is a higher-order cognitive ability that depends on coordinated activity across multiple brain networks. A single dose of a nutritional supplement may improve basic physiological status but may be insufficient to alter the connectivity efficiency of complex cognitive networks within a short period; consequently, such physiological effects may not translate into significant improvements in behavioral performance [8,56]. Although nitrate, Ginkgo biloba extract, and catechins produced favorable changes in HRV, suggesting that these supplements may provide a physiological basis for cognitive recovery by regulating autonomic function or improving cerebral blood flow, a single dose and a short observation period may be insufficient for these physiological improvements to translate into enhanced spatial localization. Furthermore, spatial localization improved during the recovery period, and a similar recovery trend was observed under the placebo condition, suggesting that the 60-minutes rest period itself may have produced a substantial natural recovery effect. Therefore, for complex cognitive tasks, acute nutritional supplementation may primarily provide potential physiological support, while any additional benefit may be limited by the extent of natural recovery.

### 4.4 Effects of different nutritional supplements on multitasking ability in esports athletes

Previous studies have indicated that although supplements such as caffeine and catechins may improve basic cognitive functions, including attention and reaction speed, their effects on complex executive functions are limited [10,12,63]. Improvements in executive function depend more strongly on long-term training that reinforces neural network connectivity than on a single dose of a supplement [57]. In addition, HF and LF/HF, as frequency-domain HRV indices, can assist in evaluating changes in autonomic regulatory patterns; however, their physiological significance should be interpreted in conjunction with other HRV indices [59,61].

The present study showed that multitasking scores after the 60-minutes recovery period were significantly higher than those in the fatigued state. However, none of the four supplements demonstrated a significant recovery effect on multitasking ability. HRV analysis showed no significant changes in HF or LF/HF under any supplementation condition, whereas RMSSD and SDNN increased to varying degrees.

These findings may be explained by the fact that multitasking ability is founded on cognitive switching and resource-allocation capacities developed through long-term training. A single dose of a supplement cannot alter established patterns of neural pathway connectivity. Furthermore, the sympathetic–parasympathetic balance supporting executive function was not significantly modulated by the short-term interventions; consequently, no significant improvement in behavioral performance was observed [10,57,63]. Although RMSSD and SDNN changed to varying degrees, HF and LF/HF did not show consistent changes. This finding suggests that partial recovery at the autonomic level may not necessarily translate immediately into improvements in complex executive function [59,61]. It should also be considered that the 60-minutes recovery period used in this study may have been insufficient to capture the full effects of some supplements on higher-order cognitive function. Although certain nutritional supplements may influence the regulation of blood flow, oxidative stress, or neurotransmitter systems, these physiological changes may not translate into improvements in complex behavioral performance within a short period. Moreover, multitasking ability improved after the recovery period, with a similar trend observed under the placebo condition. This finding further suggests that natural recovery may contribute to the restoration of higher-order cognitive abilities, whereas no additional effect of supplementation was identified.

### 4.5 Limitations

This study has several limitations. First, although the sample size was sufficient for the primary analyzes, a larger sample is required for pairwise comparisons between supplements. Moreover, given the specific characteristics of the participants, the generalizability of the findings requires further verification. Second, the administered doses differed among the supplements; future research should continue to investigate their dose–response relationships. Third, the 60-minute recovery period may have been insufficient to capture the full effects of the supplements. Fourth, the 24-hour washout period may not have been sufficient to eliminate residual effects of the supplements. Fifth, specialized equipment for measuring physiological indices was unavailable.

## 5 Conclusions

1) Mental fatigue significantly impaired esports-specific competitive performance. All four supplements significantly promoted the recovery of shooting accuracy following mental fatigue, whereas caffeine, nitrate, and catechins promoted the recovery of shooting stability. Although spatial localization and multitasking ability improved from the fatigued state to the post-supplementation state, none of the four nutritional supplements provided an additional recovery advantage over placebo for these outcomes.
2) HRV indices reflected changes in autonomic regulation in esports athletes under mental fatigue and provided complementary physiological information for evaluating the recovery process. However, the relationships between changes in HRV and the recovery of competitive performance require further investigation. In daily training, HRV indices can be used to assess the physiological status of esports athletes and support the development of more scientifically informed training plans.
3) From a practical perspective, acute nutritional supplementation may be more applicable during competitive periods in which esports athletes must maintain precise control and movement stability. For spatial localization and multitasking performance, which depend on complex cognitive processing, the appropriate scheduling of recovery time is also important. When developing recovery strategies, sport science practitioners should comprehensively consider the athlete's level of fatigue, competition duration, and the characteristics of each supplement. Acute supplementation should not be regarded as the sole strategy or as a substitute for adequate recovery.

## Data Availability

All data generated or analyzed in this study are presented in this article.
